# *Taenia solium* glutathione transferase fraction activates macrophages and favors the development of Th1-type response

**DOI:** 10.1042/BSR20181132

**Published:** 2019-01-18

**Authors:** Vera Teresa Vega-Angeles, Luis I. Terrazas, Yadira Ledesma-Soto, Lucía Jiménez, Abraham Landa

**Affiliations:** 1 Departamento de Microbiología y Parasitología, Facultad de Medicina, Universidad Nacional Autónoma de México, México 04510, CDMX; 2 Unidad de Biomedicina, Facultad de Estudios Superiores Iztacala, Universidad Nacional Autónoma de México, Tlalnepantla, Estado de México, México

**Keywords:** Classically activated macrophages (M1), cysticercosis, Glutathione transferase (GST), Th1-immune response, Taenia

## Abstract

Glutathione (GSH) transferase (GST) is an essential enzyme in cestodes for the detoxification of xenobiotics. In *Taenia solium*, two GSTs (Ts25GST and Ts26GST kDa) were isolated as a fraction (SGSTF) by GSH-Sepharose-4B. Both are located on the tegument. Immunization assays with SGSTF reduced up to 90% of the parasitic load in a murine model of cysticercosis. It prompted us to investigate how SGSTF induces this protective immune response. To test it, we exposed peritoneal macrophages to SGSTF for 24 h; such exposure favored the production of IL-12, TNF, and IL-10 as well as the expression of nitric oxide synthase 2 inducible (*Nos2*) and CD86, but did not induce the expression of chitinase-like 3 (*Chil3*). Confocal microscopy showed that the macrophages internalize the SGSTF which co-localized after 1 h with MHC-II in their plasma membranes. Macrophages exposed to SGSTF and co-cultured with anti-CD3 pre-activated T CD4^+^ cells, enhanced the proliferation of CD4^+^ cells, induced high interferon-γ (IFN-γ) secretion, and elevated the expression of CD25 and CD69, molecules associated with cell activation. Similar assay using T CD4^+^ cells from DO11.10 mice and ovalbumin (OVA) peptide+SGSTF as stimuli, showed enhanced cell proliferation and OVA-specific IFN-γ secretion. These data are in-line with those indicating that the P1, P5, and P6 peptides of *Schistosoma japonicum* 28GST highly promote T-cell proliferation and Th1 response *in vitro*. We found that such peptides are also present on Ts25GST and Ts26GST. It suggests that SGSTF activates peritoneal macrophages to a classically activated-like phenotype, and that these macrophages induce the differentiation of T CD4^+^ cells toward a Th1-type response.

## Introduction

Neurocysticercosis (NCC) is the most severe form of the diseases caused by *Taenia solium*, and remains to be the major cause of epileptic seizures in developing countries such as México. WHO estimates that approximately 2.56–8.30 million people suffer from NCC [1,2]. The early immune response against cysticercosis has been less studied due to the nature of the evolution of the disease in humans [3,4]. In contrast, it has been well characterized in a murine model of cysticercosis caused by *Taenia crassiceps* (ORF), where it is known that macrophage activation is an essential step in the development of immune responses. During the first weeks following infection, the macrophages show a classically activated phenotype, and are accompanied with a high expression of inducible nitric oxide synthase (iNOS) and interferon-γ (IFN-γ), and proliferation of T CD4^+^ cells. As the infection progresses, the macrophages exhibit an alternatively activated phenotype with the ability to block lymphocyte proliferative response and express high levels of chitinase-like 3 (Chil3) type 1 (Ym1) and arginase 1 (Arg1), likewise programmed death-ligands 1 and 2 (PD-L1 and PD-L2) [5,6]. On the other hand, it has been reported that the excreted/secreted products of the cysticerci of *T. crassiceps* (TcES) induce polarization toward Th2 responses in mice [7]. For example, dendritic cells (DC) stimulated *in vitro* with TcES are unable to respond to LPS, and in co-culture with T CD4^+^ cells from DO11.10 mice suppress IFN-γ secretion and induce IL-4 production in response to the class II (Kb)-restricted peptide epitope of ovalbumin (OVA) [8]. The carbohydrates of *T. crassiceps* also promote a Th2 response through receptors such as mannose (CD206), macrophage galactose lectin (MGL) and toll-like receptor 2 (TLR2) [9,10]. Nevertheless most of the studies have been done using TcES and only few studies have been carried out with isolated molecules in the murine cysticercosis model by *T. crassiceps*. A few such studies include: (i) the stimulation of peritoneal exudate cells (PECs) with *T. crassiceps* p66 which induces augmented NO, IFN-γ, and IL-10 secretion as well as the proliferation of lymphocytes [11]; (ii) studies with the F2 component of the *T. solium* metacestode factor (MF), and the KETc1 and KETc12 peptides which induce the proliferation of T cells and the production of IFN-γ. In addition, the last two peptides protect against murine cysticercosis [12,13].

Glutathione (GSH) transferases (GSTs) are an enzyme family whose main role is to detoxify xenobiotics by reducing GSH conjugation. Futhermore, they participate in several important processes such as the transportation of molecules, signaling, transcription, prostaglandin synthesis, formation of ion channels, isomerization, susceptibility to asthma, and in allergic reactions [14,15]. Therefore, they have been proposed as a pharmacological target for the development of anti-helminthic drugs [16]. Moreover, the GSTs used in vaccination assays have demonstrated a high degree of protection to the host. A few such instances include the protection offered by *T. solium* GST fraction (SGSTF) from *T. crassiceps* in mice, *Schistosoma japonicum* 28GST in sheep, and *Schistosoma mansoni* 28GST in humans on which a phase II clinical trial has also been performed [17,18]. Similarly, the inhibition of the activity of GSTs from Schistosomes (Sm28GST, Sh28GST and Sj28GST) using specific antibodies demonstrated a partial protective effect in animal models by affecting worm fecundity and egg viability [19,20]. In addition, studies with recombinant Sh28GST have shown that it induces a Th2 immune response [21,22]; while studies with recombinant of *Echinococcus granulosus* (EgGST) suggest that the protein induces a protective Th1-type response with the production of IgG2 and IgG3 [23,24]. Cytosolic SGSTF exists as two isoenzymes, Ts25GST and Ts26GST, with the second isoenzyme being the most abundant. Both are distributed in the cytoplasm of the parenchyma and the subtegumentary cells of the cysticerci, and are able to conjugate GSH to several electrophilic compounds [25]. It has also been observed that mice immunized with SGSTF show a decreased cysticerci burden of 90% and 85% at 4 and 8 weeks post-infection respectively, and elevated levels of specific IgG2a. In addition, the mice immunized only with the Ts26GST isoenzyme exhibited a reduced cysticerci burden of 74 and 44% at 4 and 8 weeks post-infection, respectively [26]. However, little is known about the effect of GSTs on innate immune cells such as antigen presenting cells (APCs).

In the present study, we demonstrate that *in vitro* SGSTF is capable of generating a classically activated-like phenotype in peritoneal macrophages, and these activated macrophages promote a Th1-type response in T CD4^+^ cells.

## Materials and methods

### Parasites

Cysticerci of *T. solium* were collected from a naturally infected muscle pig, provided by Facultad de Medicina Veterinaria y Zootecnia, *Universidad Nacional Autónoma de México* (UNAM).

### Mice (*Mus musculus*)

Seven to ten weeks old female BALB/c mice were purchased from Harlan Laboratories, and maintained in a pathogen-free environment at Facultad de Medicina, Universidad Nacional Autónoma de México according to the government guidelines (Official Mexican Regulation NOM-062-ZOO-1999). The protocol used here was approved by the Committee of the Ethics and Committee for Care and Management of Laboratory Animals (CICUAL) of Facultad de Medicina, UNAM.

### Infection

Seven-week-old female BALB/c mice were intraperitoneally infected with 20 cysticerci of *T. crassiceps* (ORF strain). Four months later, the cysticerci were retrieved from the peritoneal cavity, washed thrice with PBS, and immediately injected into the peritoneal cavity of naïve BALB/c mice.

### Purification of SGSTF

*T. solium* cysticerci (10 g) were homogenized, and SGSTF was separated by an affinity column of GSH-Sepharose-4B (Pharma) using the method previously described [25]. The purified SGSTF was concentrated using Amicon Ultra filter (Millipore), and its concentration and purity were tested on 12% SDS/PAGE. The SGSTF was aliquoted and stored at -20°C until further use. All solutions and materials used in this method were LPS-free.

### *In vitro* stimulation of peritoneal macrophages

PECs were elicited by thioglycollate administration into the peritoneum of BALB/c mice and collected by thorough washing of the peritoneal cavity with sterile isotonic saline solution (SSI). After determination of viability by Trypan Blue exclusion, the cells were counted and adjusted to a density of 3 × 10^6^ cells/ml in RPMI medium (Sigma) supplemented with 10% heat-inactivated FBS (Gibco) and antibiotics (100 U/ml penicillin and 100 μg/ml streptomycin, Sigma). They were then cultured on to 25-well plates at 37°C in an atmosphere of 5% CO_2_. After 2 h of incubation, the non-adherent cells were removed by washing with warm sterile SSI. Flow cytometry assays revealed that 90% of the adherent cells were F4/80+, and we denominated them peritoneal macrophages. Dose-response curves with 100, 500, 1000, and 1500 ng/ml were made to find the LPS doses where we could clearly observe the expression of activation markers of peritoneal macrophages, this was also done for the SGSTF using 0.5, 1, 5, and 10 µg (data not shown), in addition these doses were previously used in vaccination assays [26]. Therefore, peritoneal macrophages were stimulated with LPS (1 μg/ml, Sigma) or with 10 μg/ml of SGSTF to obtain M(LPS) and M(SGSTF) macrophages, respectively. Peritoneal macrophages from 8 weeks post-infection were obtained from *T. crassiceps*-infected mice M(Tc-8w) and non-stimulated macrophages control (c) were cultivated only in RPMI medium. All macrophages were cultivated for 24 h under the same conditions. This macrophage activation nomenclature was made as recently described [27].

### RNA extraction and reverse-transcription PCR

RNA was recovered from the cultured macrophages with TRIzol Reagent (Invitrogen) according to the manufacturer’s specifications. For reverse-transcription PCR (RT-PCR), 1 μg of total RNA was retro-transcribed and amplified with a couple of gen-specific primers at 50 pM using the SuperScript III One-Step RT-PCR System kit (Invitrogen). Glyceraldehyde-3-phosphate dehydrogenase (*Gapdh*) (housekeeping gene), *Arg1*, nitric oxide synthase 2 inducible (*Nos2*), and *Chil3* genes were amplified using the primers designed previously [28–30]. The amplicons were electrophoresed on 1% agarose gel and stained with Ethidium Bromide. Gels were visualized and images captured in a Kodak Electrophoresis Documentation and Analysis System (EDAS) 120. The relative transcription of genes was determined by densitometry quantitation of the bands observed for each group in the gels using ImageJ software, values are presented as arbitrary units.

### Co-culture of peritoneal macrophages with T CD4^+^ cells

Lymphocytes were obtained under sterile conditions from the spleens of naïve or 8 weeks infected BALB/c mice by perfusion, and the erythrocytes in the samples were lysed with Boyle’s solution. The T CD4^+^ cells were specifically extracted using a magnetic separation system MACS coupled to anti-CD4 antibodies (Miltenyi Biotec) according to the manufacturer’s instructions. After determination of viability by Trypan Blue assay, the T CD4^+^ cells were cultured for 2 h in 96-well flat-bottomed culture plates (Costar) previously sensitized with anti-CD3 antibodies (Biolegend). Then, non-stimulated (c), M(SGSTF), M(LPS), or M(Tc-8w) peritoneal macrophages were added in a 1:4 ratio. All co-cultures were incubated for 72 h and then the lymphocytes and supernatants were collected from them. For the proliferation assays, 0.5 μCi of tritiated thymidine (methyl-[^3^H]-TDR, Amersham) was added and the cells were incubated for a further 18 h. The cells were then harvested on a 96-well harvester (Tomtec), and finally counted using a counter 1450 microβ-plate (Trilux). Values are represented as counts/min (CPM).

### Co-culture of peritoneal macrophages with OVA-specific T CD4^+^ cells

For co-cultures stimulated with OVA peptide (323-339, Sigma), the macrophages and T CD4^+^ cells were obtained from DO11.10 mice as described before. The macrophages M(SGSTF), M(LPS), or M(Tc-8w), as well as the non-stimulated macrophages (c) were cultured with OVA peptide (5 µg/ml) for 2 h and then the T CD4^+^ cells were added in a 1:4 ratio and co-cultured for 96 h. The lymphocytes and supernatants were collected and allowed to proliferate. ELISA and flow cytometry assays were performed as described below.

### Analysis of cell surface markers

The peritoneal macrophages treated as in the section, ‘*In vitro* stimulation of peritoneal macrophages’ were harvested from culture plates using a 5 mM cold PBS-EDTA mixture for 10 min, washed, and then re-suspended in an FACS buffer (BD Biosciences). To avoid unspecific unions with antibodies, anti-mouse CD16/32 (93, dil. 2:100) was added for 20 min at 4°C to block the Fc receptors on the cell surface of macrophages. Then cells were washed with FACS buffer (BD Biosciences) and stained as indicated, with antibodies against F4/80 (BM8, 1:100), MHC-II (M5/114.15.2, 1:100), CD206 (C068C2, 2:100), IL-4Rα (I015F8, 4:100), PD-L1 (B7-H1, 2:100), PD-L2 (TY-25, 2:100), CD80 (16-10A1, 4:100), and CD86 (GL-1, 2:100) in staining buffer at 4°C and used according to manufacturer’s instructions (Biolegend). The stained cells were analyzed on a FACsCalibur flow cytometer (BD Biosciences) using Cell Quest and FlowJo software. Parallel samples of the cells were stained with the corresponding isotype control. T CD4^+^ cells obtained from the section, ‘Co-culture of peritoneal macrophages with lymphocyte T cells’ were washed with the FACS buffer (BD Biosciences), marked with anti-CD4 (GK1.5, 1:100), anti-CD25 (3C7, 1:100) and anti-CD69 (H1.2F3, 1:100) antibodies (Biolegend), and analyzed on Attune NxT flow cytometer (Thermo Fisher) using FlowJo software.

### Confocal microscopy

Peritoneal macrophages (2 × 10^6^ cells/ml) were cultured on to a sterile slide cover treated with poly-l-lysine (Neuvitro) in 24-well plates (Costar) either without or stimulated with SGSTF (10 μg/ml) for 15, 30, and 60 min. Then, cells were washed, fixed in 4% paraformaldehyde and treated with 0.1% Triton X-100 in PBS for 20 min and non-specific background staining was blocked with 5% BSA (Sigma-Aldrich) diluted in 0.1 M PBS (pH 7.2). Subsequent incubation with primary antibodies against SGSTF (1:200) and IA-IE (1:100, MHC-II) (Biolegend) were performed at 4°C overnight. Secondary antibodies coupled with FITC (1:100) and Texas Red (1:100) were used respectively (Santa Cruz Biotechnology). Slides were mounted on to UltraCruz Hard-set mounting with DAPI medium (Santa Cruz Biotechnology) until observation. The samples were observed under an LMS 800 Zeiss microscope at 63× and analyzed using ZEN Zeiss and ImageJ software.

For experiments on no-SGSTF internalization, cytochalasin D treatment of macrophages was carried out as described earlier [31]. Briefly, macrophages were incubated with cytochalasin D (10 µM, Sigma-Aldrich) for 30 min at 37°C. Cytochalasin D was removed by two washes with PBS, then was incubated with SGSTF for 30 min and following the treatment described above. Macrophages without cytochalasin were used as control. The orthogonal images were taken in projections of 17-18 sequential sections, were scanned five to seven times each, with a thickness of 10.5 µm and captured with the objective 63× with immersion oil and a zoom of 1.5 in a Leica DMi8 SP8 microscope and processed with their software.

### Cytokine assays

Concentrations of IL-10, IL-12, IL-4, IFN-γ and TNF were measured in the collected supernatants from all cultures using the ELISA Development kit (PeproTech) in 96-well plates (NuncMaxiSorp) following the protocol described by the manufacturer.

### GSTs primary sequence analyses

An alignment of the primary sequences of the three peptides, P1, P5 and P6 from *S. japonicum* 28GST was performed using the Clustal W program (http://www.ebi.ac.uk/clustalw) with those of the *T. solium* 25GST and 26GST.

### Statistical analysis

One-way ANOVA with Bonferroni’s test were used for the statistical analyses of collected data and *P*-values <0.05 were considered significant.

## Results

### Macrophages exposed to SGSTF express M1 activation markers

**Figure 1:**
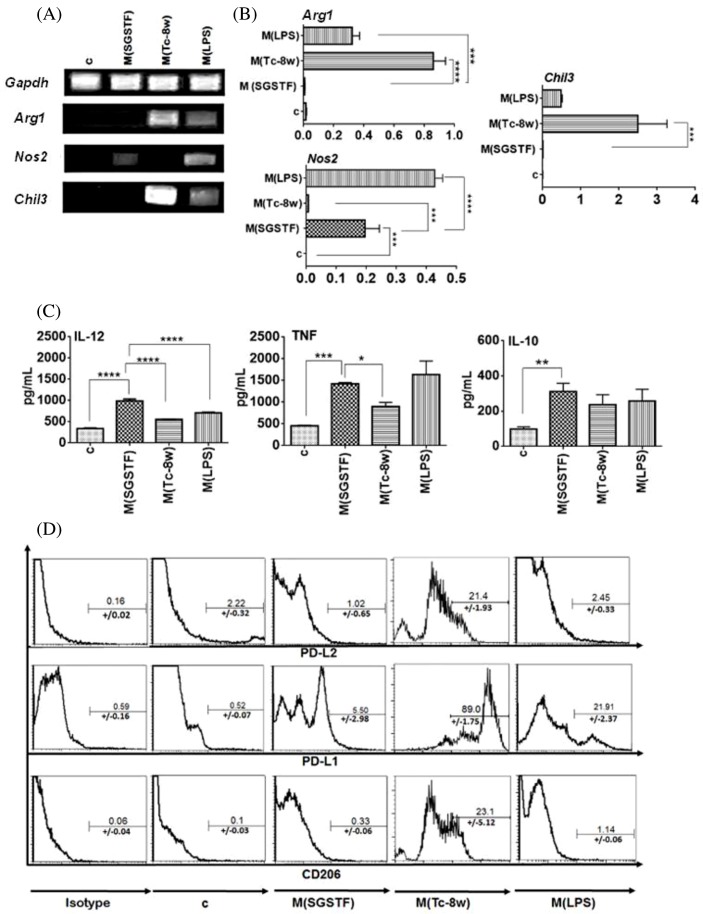
Effect on the expression of classically and alternative activation markers on peritoneal macrophages by the *in vitro* stimulation for 24 h with SGSTF (**A**) Determination of transcript levels of *Gapdh, Arg1, Nos2*, and *Chil3* by RT-PCR. (**B**) Relative transcription in arbitrary units. (**C**) Determination of cytokines (TNF, IL-12, IL-10) in the culture supernatants from all groups of macrophages by ELISA. (**D**) Flow cytometry to determine PD-L2, PD-L1, and CD206 on macrophage membranes. In (C) control macrophages of non-stimulation, in SGSTF macrophages stimulated with SGSTF (10 μg/ml) and in M(LPS) and M(Tc-8wk) macrophages controls of classically or alternative activation respectively. The data shown are representative of three independent experiments (*n*=3) and are expressed as mean  ±  S D. One-way ANOVA with Bonferroni’s post hoc test were used for statistical analyses (*P<0.05, **P<0.005, ***P<0.0005, ****P<0.0001).

In order to determine whether SGSTF may directly activate peritoneal macrophages, we exposed them to SGSTF for 24 h and compared the expression of several markers to those obtained from the non-stimulated control (c), M(LPS), and M(Tc-8w) macrophages. Figure 1A,B shows the results of the RT-PCR assay carried out to measure the expression levels of the classical and alternative activation gene markers. M(SGSTF) macrophages expressed *Nos2*, but did not express *Arg1* and *Chil3*. In contrast, the M(Tc-8w) macrophages expressed high levels of *Arg1* and *Chil3*, but did not express *Nos2*. In addition M(LPS) macrophages highly expressed *Nos2* and showed moderate expression of *Arg1* and *Chil3*. The non-stimulated macrophages (c) did not express any of the genes tested. *Gapdh* gene was used as a housekeeping gene and it presented a constant rate of expression in all macrophages groups. Moreover, supernatants from the same cultures were tested for cytokine production. Figure 1C shows the determination of the proinflammatory cytokines secreted by macrophages in culture by ELISA. Macrophages exposed to SGSTF displayed significantly higher production of IL-12 compared with the non-stimulated (c), to M(Tc-8w) and to M(LPS) macrophages, while TNF production was higher in M(SGSTF) and M(LPS) macrophages than non-stimulated (c) and M(Tc-8w) macrophages. Interestingly, secretion of IL-10 increased significantly in M(SGSTF) macrophages with respect to non-stimulated macrophages (c) and there were no differences with respect to M(LPS) and M(Tc-8w). In addition, flow cytometry assays showed that SGSTF-exposed macrophages expressed low levels of PD-L1 that was no statistically significant and did not express PD-L2 or CD206 in a manner similar to M(LPS) macrophages (Figure 1D). As expected, M(Tc-8w) macrophages displayed a typically high expression of PD-L1, PD-L2, and CD206 [32] and non-stimulated macrophages (c) did not express any of these markers.

### Expression of co-stimulatory molecules and MHC-II on peritoneal macrophages exposed to SGSTF for 24 h

**Figure 2:**
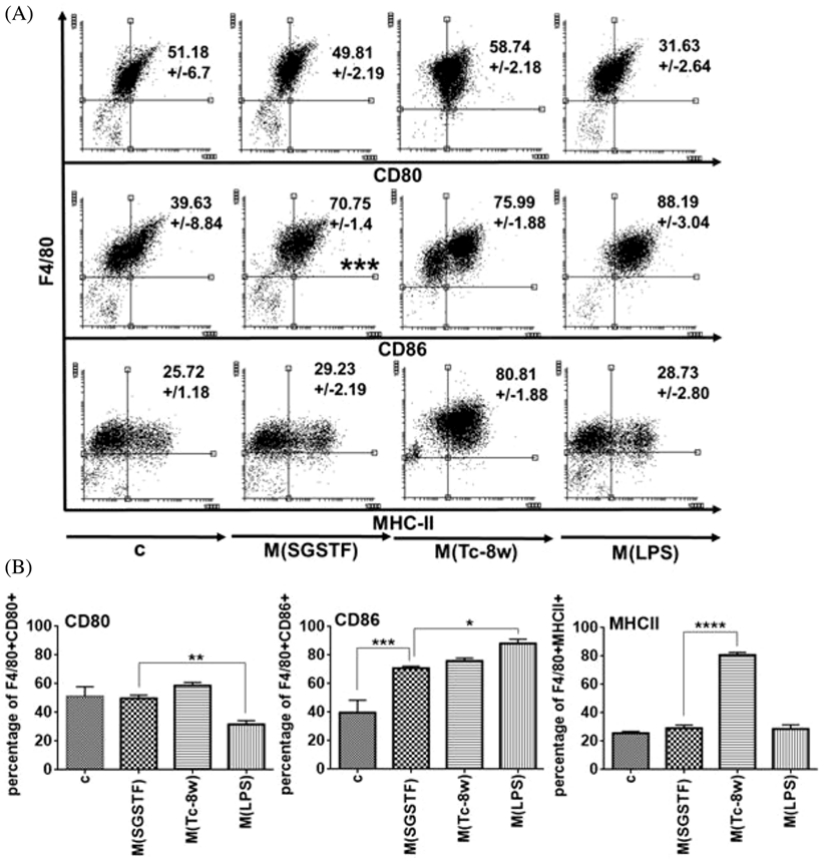
Flow cytometry assays to detect expression of CD80, CD86, and MHC-II surface molecules in peritoneal non-stimulated and SGSTF-stimulated macrophages (**A**) Dot-plots show the expression of CD86 in non-stimulated control macrophages (c) and in SGSTF-stimulated macrophages. M(Tc-8w) and M(LPS) peritoneal macrophages were used as controls for alternative or classical activation. (**B**) Bar graphs show the percentage of double positive cells for CD80, CD86, or MHC-II accordingly. Data shown are representative of three independent experiments and are expressed as mean  ±  S.D. (*n* = 3). One-way ANOVA with Bonferroni’s post hoc test were used for statistical analyses (*P<0.05, **P<0.005, ***P<0.0005, ****P<0.0001).

Flow cytometry assays were performed using specific antibodies against CD80, CD86, and MHC-II molecules to determine their expression in the macrophages after stimulation for 24 h (Figure 2A,B). CD80 was highly expressed in the M(Tc-8w) macrophages followed by the SGSTF-stimulated macrophages and non-stimulated macrophages (c); but they did not show significant differences. However, expression of CD80 in M(SGSTF) macrophages was significantly higher than in M(LPS) macrophages, but M(SGSTF) macrophages did not show differences with respect to the non-stimulated macrophages. Furthermore, CD86 expression was significantly higher in M(SGSTF) macrophages with respect to non-stimulated macrophages (c) and it was comparable with respect to M(Tc-8w), but it was less than M(LPS) macrophages. In addition, MHC-II in M(SGSTF) macrophages showed no differences in expression with respect to non-stimulated (c) and M(LPS) macrophages. In contrast, M(Tc-8w) macrophages showed higher MHC-II expression with respect to others.

### Uptake of SGSTF by macrophages

**Figure 3:**
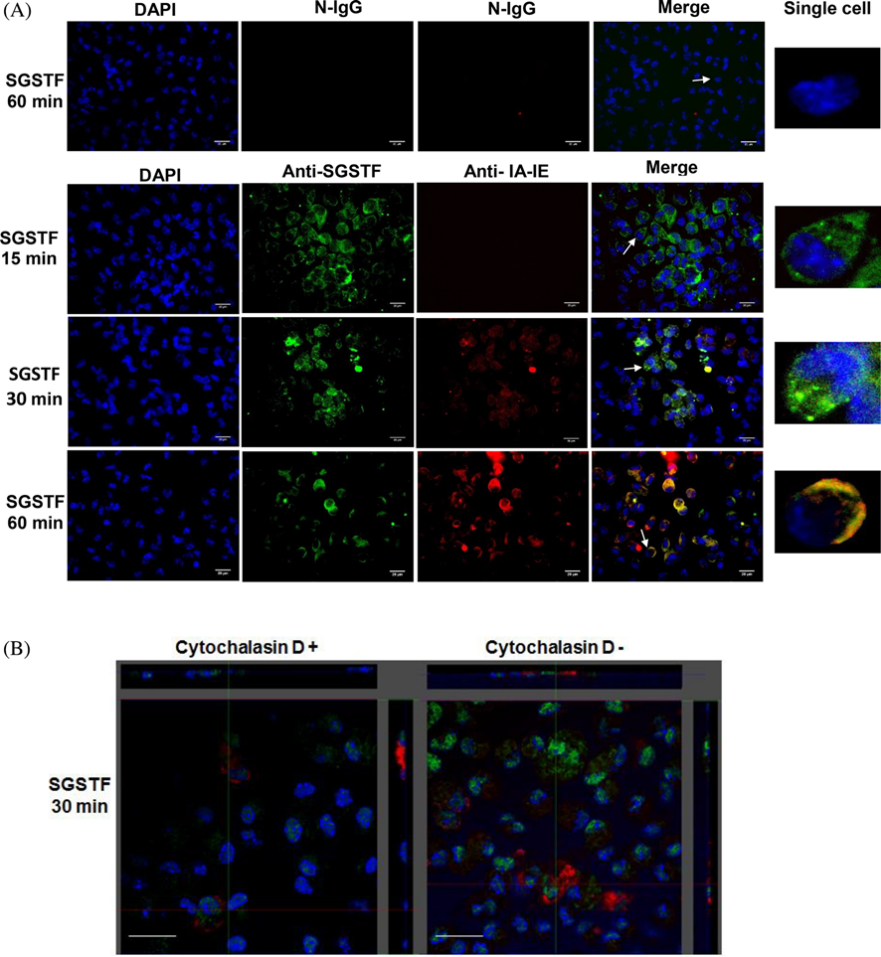
Uptake of SGSTF by peritoneal macrophages as observed by confocal microscopy (**A**) Macrophages stimulated with SGSTF (SGSTF) for 15, 30, and 60 min were initially incubated with N-IgG or antibodies against SGSTF (anti-SGSTF) and against IA-IE (Anti-IA-IE). Secondary antibodies conjugated to FITC (green) and Texas Red (red) respectively, were used to identify SGSTF and MHC-II. Nuclei from macrophages were stained with DAPI. We depict the merged images and digital amplifications of a single cell (white arrow) to observe the three channels in detail. Scale bars in figures represent 20 μm. (**B**) Orthogonal images (single Z-plane) from macrophages incubated without (−) and with (+) cytochalasin D and stimulated with SGSTF for 30 min, and following the treatment mentioned above.

In order to determine whether SGSTF was being internalized by macrophages we performed a confocal microscopy assay (Figure 3), where macrophages were exposed to SGSTF for 15, 30, and 60 min. At 15 min, a strong green signal (SGSTF) was found dispersed in the cytoplasm and MHC-II was detected as a weak red signal in some cells; the merged image at this time point displayed only SGSTF in the cytoplasm. After 30 min, most of the SGSTF was concentrated in granules, although some was still dispersed in the cytoplasm. However, it is noteworthy to mention that at this time point, MHC-II (red signal) started to appear in cytoplasm; the merged image at this time point displayed a poor co-localization between SGSTF and MHC-II. At 60 min, a major part of the SGSTF appeared concentrated closely to the membrane in most macrophages, and MHC-II increased in intensity and showed the same pattern as SGSTF. The merge image at this time point showed a crescent-shaped co-localization between SGSTF and MHC-II (yellow signal) in most of the macrophages. However, some cells only showed green (SGSTF) or red (MHC-II) signals without any co-localization. This could be explained by the lack of synchronization amongst macrophages in culture. Normal IgG (N-IgG) was used as a control; it showed non-unspecific recognition of the primary and secondary antibodies and no signal was observed. Orthogonal images of experiments performed incubating macrophages without and with cytochalasin D and after incubating for 30 min with SGSTF (Figure 3B). Macrophages without cytochalasin D showed similar internalization of SGSTF as observed at the same time in Figure 3A (SGSTF 30 min) with green signal on to cytoplasm and a weak red signal to MHC-II. In contrast, cytochalasin D treated macrophages decrease the internalization of SGSTF. Noteworthy, we also observed the delay in the appearance of the MHC-II molecule in macrophages treated with cytochalasin D. Orthogonal analysis of these experiments shown that the SGSTF is inside macrophages.

### Macrophages exposed to SGSTF favor IFN-γ production in T CD4^+^ cells

**Figure 4:**
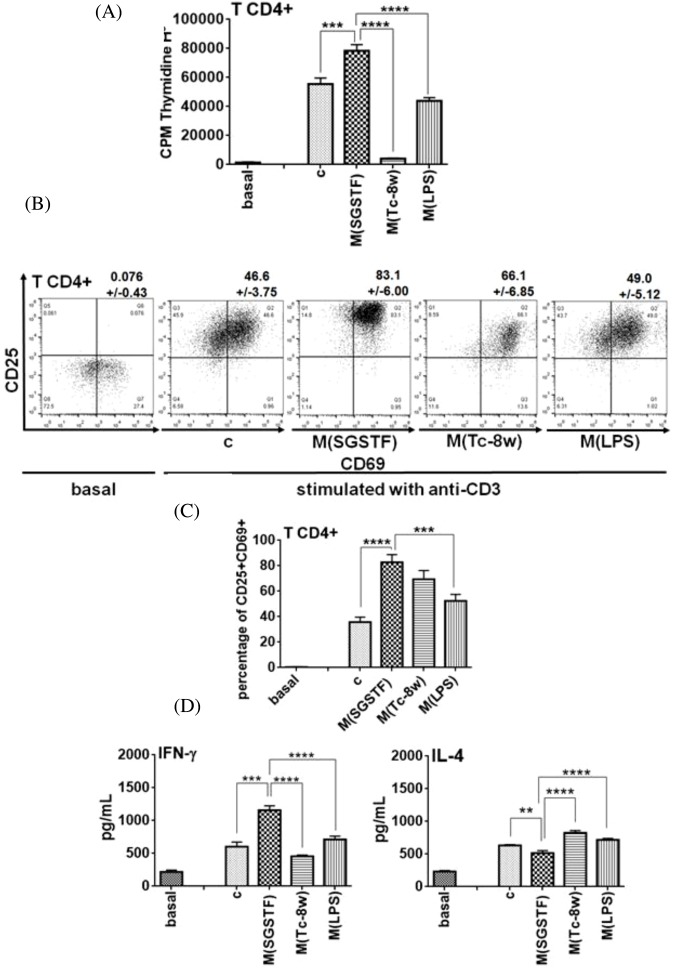
Response of naive T CD4^+^ cells pre-treated with anti-CD3 antibody co-cultured with (c) non-stimulated, M(SGSTF), M(Tc-8w), and M(LPS) macrophages during 72 h (**A**) Proliferation, (**B**,**C**) Expression of CD69 and CD25 in T CD4^+^ cells. (**D**) Cytokines secreted (IFN-γ and IL-4) by co-cultures. T CD4^+^ cells not treated with anti-CD3 and co-cultured with non-stimulated macrophages were used as basal control (basal). Data shown are representative of three independent experiments and are expressed as mean  ±  S.D. (*n*=3). One-way ANOVA with a Bonferroni’s test were used for statistical analyses (**P<0.005, ***P<0.0005, ****P<0.0001).

Then, we analyzed the ability of the SGSTF-stimulated macrophages to modulate T CD4^+^ cell responses. Thus, we performed different assays with anti-CD3 pre-activated CD4^+^ cells that were co-cultured for 72 h with macrophages exposed to different stimuli. Figure 4A shows the macrophages influence on T CD4^+^ cell proliferation; the CD4^+^ cells co-cultured with SGSTF-exposed macrophages displayed a significantly higher proliferation compared with co-cultures with non-stimulated (c) and M(LPS) macrophages. As expected by previous reports, the CD4^+^ cells co-cultured with M(Tc-8w) macrophages displayed a markedly inhibited cell proliferation, and the T CD4^+^ cells co-cultured without anti-CD3 antibodies showed no proliferation (basal). In addition, T CD4^+^ cells from parallel co-cultures were analyzed by flow cytometry to detect the expression of CD69 and CD25 molecules, which are associated with cell activation, SGSTF-exposed macrophages showed a pronounced augmented expression of CD69 and CD25 on CD4 cells (Figure 4B,C) compared with T CD4^+^ cells co-cultured with non-stimulated (c) or M(LPS) macrophages and no significant differences with respect to M(Tc-8w) macrophages were found. Finally, a basal expression of CD69 and CD25 was observed in co-cultures with T CD4^+^ cells not treated with anti-CD3 and with non-stimulated macrophages (basal).

Supernatants from these co-cultures were analyzed by ELISA for IFN-γ and IL-4 production (Figure 4D). Co-cultured T CD4^+^ cells with SGSTF-exposed macrophages displayed a significant increase in IFN-γ production with respect to those co-cultured with non-stimulated macrophages (c) and this increment was also significant with respect to M(LPS) and M(Tc-8w) macrophages. Interestingly, IL-4 secretion by T CD4^+^ cells co-cultured with the SGSTF-exposed macrophages was significantly lower compared with T CD4^+^ cells co-cultured with M(Tc-8w) and with M(LPS) macrophages and with non-stimulated macrophages (c). All the co-cultures with anti-CD3 pre-activated T CD4^+^ cells and different groups of macrophages showed IL-12 concentrations lesser than 300 pg/ml (data not shown). Co-cultures with non-stimulated macrophages and no pre-CD3 activation showed the lowest concentration of all measured cytokines (basal).

**Figure 5:**
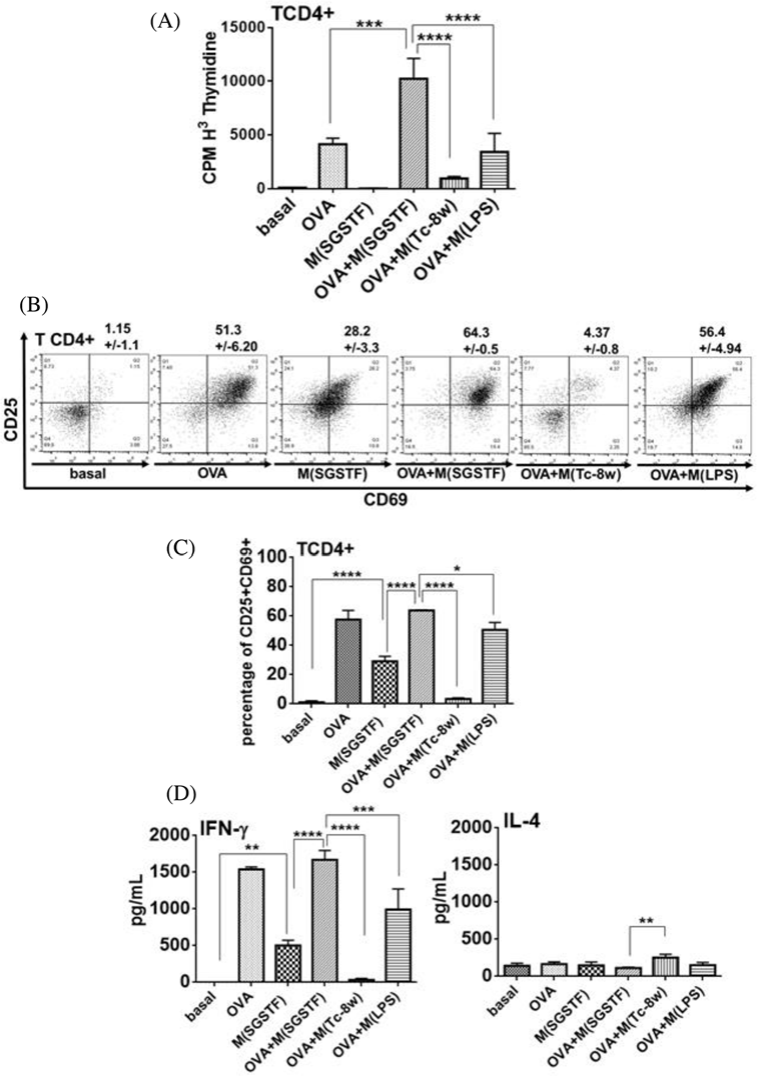
Response of transgenic T CD4^+^ cells (DO11.10) in 72 h co-cultured with macrophages: non-stimulated without OVA peptide (basal) and non-stimulated with OVA (OVA, 10 μg/ml) M(SGSTF) macrophages and M(SGSTF) macrophages with OVA peptide (OVA+M(SGSTF)); M(Tc-8w) macrophages plus OVA (OVA+M(Tc-8w)) and M(LPS) macrophages plus OVA (OVA+M(LPS)). (**A**) Proliferation; (**B**,**C**) expression of CD69 and CD25 in T CD4+ cells. (**D**) Cytokines secreted (IFN-γ, and IL-4) in co-culture supernatants were detected by ELISA. Data shown are representative of three3 independent experiments and are expressed as mean  ±  S.D. (*n*= 3). One-way ANOVA with Bonferroni were used for statistical analyses (*P<0.05, **P<0.005, ***P<0.0005, ****P<0.0001).

In order to demonstrate that the effect of SGSTF on macrophages may also be an antigen-specific response, we performed similar experiments as described above, but using purified DO11.10 T CD4+ cells, which express a TCR specific for OVA peptide 323-339. Co-cultures with SGSTF-stimulated macrophages showed a remarkable and significantly higher proliferation rate of DO11.10 T CD4+ cells when OVA peptide was added to the co-cultures (OVA+M(SGSTF)) compared with non-stimulated macrophages plus OVA peptide (OVA) and to M(LPS) macrophages plus OVA peptide. In contrast, T CD4+ cells co-cultured with M(Tc-8w) macrophages displayed a limited proliferation. No proliferation was observed in co-cultures with non-stimulated (basal) and M(SGSTF) macrophages without OVA peptide (Figure 5A).

In addition, Figure 5B,C show the expression of CD69 and CD25 detected in the co-cultured DO11.10 T CD4^+^ cells. Co-cultures with SGSTF-stimulated macrophages plus OVA peptide (OVA+M(SGSTF)) showed no significant differences in CD69 and CD25 expression with respect to co-cultures with non-stimulated macrophages plus OVA peptide (OVA), but it was significant with respect to the co-cultures with M(LPS) macrophages plus OVA (OVA+M(LPS). In contrast, T CD4^+^ cells co-cultured with M(Tc-8w) macrophages plus OVA peptide showed a decrease in the expression of these molecules with respect to SGSTF-stimulated macrophages plus OVA peptide (OVA+M(SGSTF)). Interestingly, T CD4+ cells cultured with SGSTF-stimulated macrophages without OVA exhibited a higher expression of these molecules than co-cultures without stimulus (basal) and with respect to M(Tc-8w) macrophage plus OVA peptide. Furthermore, supernatants from all these co-cultures were analyzed for the production of IFN-γ and IL-4. As observed in Figure 5D, T CD4+ cells co-cultured with SGSTF-exposed macrophages plus OVA peptide showed high production of IFN-γ (OVA+SGSTF) which was higher than the co-cultures with M(LPS) macrophages, however, this level was not statistically different with respect to co-cultured T CD4+ cells only stimulated with OVA. In contrast, T CD4+ cells co-cultured with M(Tc-8w) macrophages plus OVA showed no secretion of IFN-γ comparable with T CD4+ cells co-cultured with non-stimulated macrophages and without OVA (basal). Interestingly, co-cultures SGSTF-stimulated macrophages without OVA showed a IFN-γ secretion (M(SGSTF)) that was significant with respect to basal. In contrast, IL-4 secretion by T CD4+ cells were less than 300 pg/ml in all co-cultures and only significantly differences were found amongst OVA+M(Tc-8wk) and M(SGSTF)+OVA co-cultures.

### Search for Th1 epitopes’ localization in *Taenia solium* GST

**Figure 6:**
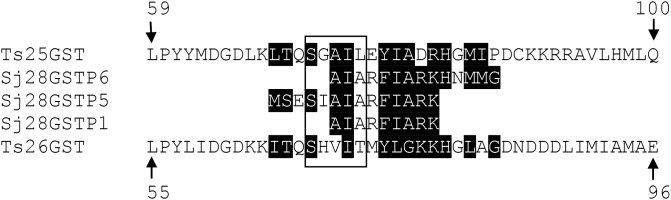
Alignment of the primary sequences of the peptides (P1, P5, and P6) of *S. japonicum* 28 kDa GST (Sj28GST; P26624) with SGSTFs of 25 kDa (Ts25GST; AAM64054) and 26 kDa (Ts26GST; AAX19694) Homology is shown in white lettering with a black background. In a closed box are shown the amino acids that form the consensus SNAIL/TRAIL motif.

To ascertain whether the peptides P1, P5, and P6 (Th1 epitopes) from *S. japonicum* 28GST were also localized on the *T. solium* 25GST and 26GST [33], their primary sequences were aligned to find similarities (Figure 6). Our analysis of the alignment indicated that the three peptides (P1, P5, and P6) of *S. japonicum* 28GST share a homology in the range of 64.31, 71.42, and 77.77% with the *T. solium* 25GST and 26GST. Interestingly, these peptides are localized either inside or around of the conserved motif (SNAIL/TRAIL) presented in all GSTs [34].

## Discussion

The innate immune system is the first line of defense against various pathogens and is mediated by cells such as macrophages and dendritic cells, which possess different classes of pattern recognition receptors (PRRs, such as Toll-like receptors) to recognize pathogen-associated molecular patterns (PAMPs). In murine (BALB/c) cysticercosis caused by *T. crassiceps*, macrophages and T CD4^+^ cells are the major players that modulate the Th1/Th2 balance and the parasite growth [35,36]. It is known that the depletion of alternatively activated macrophages during early infection in this model decreases parasitic burden and restores the antigen-specific proliferative response in T lymphocytes from late infections [6,32]. In contrast, using a C57BL/6-resistant mouse strain, which maintains the presence of classically activated macrophages and also a pro-inflammatory profile throughout the infection, restricts the growth of this parasite [37]. These reports suggest that macrophages are important to develop Th1-responses to protect mice from cysticercosis.

Previously, we have demonstrated that immunization with 5–10 µg of SGSTF induces a reduction in up to 90% in parasite burden in experimental cysticercosis [26]. However, immunological mechanism associated with SGSTF protection was undefined. To test if SGSTF and macrophages may interact to modulate Th1/Th2 immune responses *in vitro*, we exposed macrophages to SGSTF for 24 h. These macrophages expressed the *Nos2* and favored TNF and IL-12 production but did not express alternatively activated macrophages’ markers such as the *Arg1* and *Chil3*, PD-L2, CD206, or IL-4Rα, suggesting that stimulation with SGSTF induces macrophages toward a pro-inflammatory profile. These results are in agreement with observations that macrophages activated by LPS, IFN-γ, TNF, or macrophages from early stages of infection in the murine model of *T. crassiceps* cysticercosis highly express the same pro-inflammatory cytokines but not alternative activation markers and because of that are considered classically activated macrophages [36,38,39]. However, secretion of IL-10 was also detected in SGSTF-stimulated macrophages and it is known that alternatively activated macrophages are sources of IL-10, a cytokine that promotes homeostasis, suppresses the pro-inflammatory response, limits the damage, and is involved in tissue remodeling and the clearance of apoptotic cells [40,41]. The presence of IL-10 in SGSTF-stimulated macrophages could be indicating that they are responding to SGSTF in a mixed way. Nevertheless SGSTF-stimulated macrophages secreted greatest concentrations of IL-12 than IL-10 and the ratio of IL-12/IL-10 that has been used to determine the classical or alternative activation in macrophages [42,43] indicates that dominance of IL-12 over IL-10 in M(SGSTF) macrophages favors the pro-inflammatory phenotype, thus SGSTF induces in this way an M1-like phenotype. In addition the evidence of a recent report that demonstrated that M(Tc-8w) macrophages which arises from the late stages of infection in the cysticercosis model by *T. crassiceps* are mostly PD-L2^+^ and do not express IL-10 [44] showed that in this infection, presence of IL-10 is not an alternative activation determinant, but membrane markers as PD-Ls and the diminished expression of pro-inflammatory cytokines markers are more relevant [37]. Furthermore, SGSTF-stimulated macrophages showed a high and sustained expression of CD86, co-stimulatory signals through B7 molecules are crucial for the activation of APCs and the proliferation and cytokine production of T cell; the absence of this signal induces clonal anergy when TCR stimulation is present [45,46]. It is known that CD86 and CD80 are ligands for CD28 and are related to the development of Th1 or Th2 responses when APCs prime naïve T cells and have different roles in various diseases [47,48]. In addition, the decreased levels of CD80 and CD86 in *T. crassiceps*-susceptible mice [37], and the increment in CD86 levels in SGSTF-stimulated macrophages together suggest the importance of CD86 in the development of Th1 response. Additionally, macrophages that uptake SGSTF in culture, show a co-localization between MHC-II and SGSTF after 60 min; which suggests that an early expression of the MHC-II molecule is involved in antigenic presentation to T cells. All these findings indicate that macrophages respond to SGSTF as an inflammatory stimulus and express molecules that are considered markers for classically activated macrophages such as Nos2, IL-12, and TNF and similarly the molecules involved in the antigen presentation to T CD4+ cells, such as CD86, MHC-II, and MHC-II+SGSTF complex.

Having demonstrated that SGSTF exposure drives peritoneal macrophages toward an M1-like phenotype, our next step was to analyze if such activation may impact the differentiation of naïve T cells toward the Th1 phenotype and the expansion and survival of activated T cells [49,50]. Co-cultures with SGSTF-stimulated macrophages promoted augmented proliferation of T CD4^+^ cells and augmented expression of CD25 and CD69 molecules. It is known that CD25 and CD69 on the surface of activated lymphocytes in humans and mice are crucial for the amplification of IL-2 signaling, for the secretion of TNF and to elicit Th1 and Th17 responses [51,52]. Co-cultures with SGSTF-stimulated macrophages also showed an increase in the production of IFN-γ and a decrease in IL-4 secretion with respect to co-cultures with M(Tc-8w) macrophages that suppressed the proliferation of T CD4^+^, even when expression of CD25 and CD69 is present. It has been observed that administration of rIFN-γ in mice has a protective role in murine cysticercosis model, on the contrary, anergy of Th1 cells and dominance of Th2-type response are permissive for this parasite [53]. According to these previous data, the reported protective effect of SGSTF on experimental cysticercosis [25,26] may be due to activating directly innate cells such as macrophages where an M1-like phenotype is induced that in turn favors Th-1 polarization that is protective in this experimental infection. In contrast, if macrophages are eliminated at an early stage of infection, a permissive Th2-type response is established with a high production of IL-4 and a low production of IFN-γ, which favors the parasite growth [32,53]. The high production of IFN-γ together with the expression of CD25 and CD69 by T CD4^+^ cells and higher rates of proliferation induced by the SGSTF-stimulated macrophages, support the idea that pro-inflammatory activation of macrophages by SGSTF is driving T CD4^+^ cells toward a Th1 phenotype. In addition, the production of IL-12 by the SGSTF-stimulated macrophages, is in agreement with studies showing that IL-12 is an inductor of IFN-γ secretion, and necessary to develop a Th1 response against helminths, including *T. crassiceps* [54,55].

On the other hand, in a similar assay using T CD4^+^ cells from DO11.10 mice and OVA+SGSTF peptide as stimuli, we detected enhanced cell proliferation and OVA-specific IFN-γ secretion, but no significant differences in the expression of CD25 and CD69 markers, which suggests that SGSTF favor the pro-inflammatory response to an unrelated antigen such as OVA. This is important because previous studies have demonstrated that *T. crassiceps* antigens induce secretion of IL-4 and IL-10 and inhibited the proliferation and IFN-γ response against OVA in DO11.1 T CD4^+^ cells [5,8,9] likewise similar effects were also demonstrated in other helminth antigens [56,57]. Furthermore, SGSTF stimulus in co-cultures showed IFN-γ secretion and activation markers in OVA-specific T CD4 cells, this result supports the idea that SGSTF is acting as an adjuvant that drives macrophages to prime T CD4^+^ cells to a pro-inflammatory profile.

Recently, vaccination assays have demonstrated that nine Th1-type peptides of *S. japonicum* 28GST promote T-cell proliferation and a Th1 response *in vitro*. Peptide 6 strongly promotes a Th1 response with a high production of IFN-γ and IL-2, and has been proposed as an antigen that can be targetted to develop a vaccine against *S. japonicum* infection [33]. It is of note that these peptides (P1, P5, and P6) promote strong Th1 responses against *S. japonicum*, are also presented in the *T. solium* 25GST and 26GST, and are localized in the consensus conserved motif (SNAIL/TRIAL) that distinguishes GST parasites from mammalians [34]. In addition, these findings suggest that SGSTF together with other GSTs could be used to develop a polyvalent vaccine against helminths or to develop a *Taenia* vaccine alone or together with other molecules such as p66, F2 metacestode factor, GK1 and GK2 peptides [11–13]. However, the mechanisms that induce T CD4^+^ cells toward Th1 or Th2 responses have not been solved yet in cysticercosis, but discovery of new factors such as natural antigens and the mechanism of how antigen-producing classically activated (M1) or alternatively activated (M2) macrophages function are important to understand the polarization of immune response.

In conclusion our data provide evidence that SGSTF is able to bind and activate macrophages to an M1-like phenotype with increased expression of co-stimulatory molecules as well as soluble factors (IL-12 and TNF) that are able to promote in T CD4^+^ murine cells a Th1-type response *in vitro*, which may be associated with the conferred protection against cysticercosis previously reported for this molecule.

## Abbreviations

APC: antigen presenting cell
Arg1: arginase 1
Chil3: chitinase-like 3
CONACYT: Consejo Nacional de Ciencia y Tecnología
Gapdh: glyceraldehyde-3-phosphate dehydrogenase
GSH: glutathione reduced
GST: GSH transferase
IFN-γ: interferon-γ
IL: interleukin
NCC: neurocysticercosis
Nos2: nitric oxide synthase 2 inducible
OVA: ovalbumin
PD-L: programmed death-ligand
PEC: peritoneal exudate cell
rIFN-γ: recombinant INF-γ
RT-PCR: reverse-transcription PCR
SGSTF: *Taenia solium* GST fraction
SSI: sterile isotonic saline solution
TcES: excreted/secreted products of the cysticerci of *Taenia crassiceps*
TCR: T cell receptor
TNF: tumor necrosis factor
UNAM: *Universidad Nacional Autónoma de México*
